# No-shows to primary care appointments: subsequent acute care utilization among diabetic patients

**DOI:** 10.1186/1472-6963-12-304

**Published:** 2012-09-06

**Authors:** Lynn A Nuti, Mark Lawley, Ayten Turkcan, Zhiyi Tian, Lingsong Zhang, Karen Chang, Deanna R Willis, Laura P Sands

**Affiliations:** 1 School of Nursing, College of Health and Human Sciences, Purdue University, 502 N. University Street, West Lafayette, IN, 47907-2069, USA; 2 Weldon School of Biomedical Engineering, Purdue University, 206 S. Martin Jischke Drive, West Lafayette, IN, 47907-2032, USA; 3 Department of Mechanical and Industrial Engineering, Northeastern University, 360 Huntington Avenue, Boston, MA, 02115, USA; 4 Regenstrief Center for Healthcare Engineering, Purdue University, 203 Martin Jischke Drive, West Lafayette, IN, 47907, USA; 5 Department of Statistics, Purdue University, 150 N. University Street, West Lafayette, IN, 47907, USA; 6 Department of Family Medicine, Indiana University School of Medicine, 1110 W Michigan Street, Long Hall, Suite 200, Indianapolis, IN, 46202, USA; 7 School of Nursing, College of Health and Human Sciences, Center for Aging and the Life Course, Purdue University, 502 N. University Street, West Lafayette, IN, 47907-2069, USA

**Keywords:** No-show, Diabetes, Emergency department visits, Hospital admissions, Outcomes

## Abstract

**Background:**

Patients who no-show to primary care appointments interrupt clinicians’ efforts to provide continuity of care. Prior literature reveals no-shows among diabetic patients are common. The purpose of this study is to assess whether no-shows to primary care appointments are associated with increased risk of future emergency department (ED) visits or hospital admissions among diabetics.

**Methods:**

A prospective cohort study was conducted using data from 8,787 adult diabetic patients attending outpatient clinics associated with a medical center in Indiana. The outcomes examined were hospital admissions or ED visits in the 6 months (182 days) following the patient’s last scheduled primary care appointment. The Andersen-Gill extension of the Cox proportional hazard model was used to assess risk separately for hospital admissions and ED visits. Adjustment was made for variables associated with no-show status and acute care utilization such as gender, age, race, insurance and co-morbid status. The interaction between utilization of the acute care service in the six months prior to the appointment and no-show was computed for each model.

**Results:**

The six-month rate of hospital admissions following the last scheduled primary care appointment was 0.22 (s.d. = 0.83) for no-shows and 0.14 (s.d. = 0.63) for those who attended (*p* < 0.0001). No-show was associated with greater risk for hospitalization only among diabetics with a hospital admission in the prior six months. Among diabetic patients with a prior hospital admission, those who no-showed were at 60% greater risk for subsequent hospital admission (HR = 1.60, CI = 1.17–2.18) than those who attended their appointment. The six-month rate of ED visits following the last scheduled primary care appointment was 0.56 (s.d. = 1.48) for no-shows and 0.38 (s.d. = 1.05) for those who attended (*p* < 0.0001); after adjustment for covariates, no-show status was not significantly related to subsequent ED utilization.

**Conclusions:**

No-show to a primary care appointment is associated with increased risk for hospital admission among diabetics recently hospitalized.

## Background

Diabetes is a rising health care concern as its prevalence continues to grow in the United States. Diabetes mellitus was ranked as the seventh leading cause of death in 2009 [1]. In 2007, $218 billion was spent in total estimated costs for diabetes, with $153 billion due to medical costs and $65 billion due to reduced productivity [2]. There were nearly 125 million visits to Emergency Departments (ED) in 2008 and over 98.5 million were for adults; the most frequent reasons for adult ED visits revealed that diabetes mellitus without complications ranked third (9.3%) of all ED visits [3]. In 2007, an estimated $3.87 billion was attributed to emergency department costs for diabetes [4]. In 2008 over 7.7 million hospital admissions and $83 billion in costs were attributed to diabetes [5].

Complications and costs from diabetes can be reduced by consistent and effective disease management [6]. For effective diabetes management, national clinical practice guidelines recommend patients visit their healthcare provider every 3 to 6 months [6]. For a majority of patients, the primary care provider (PCP) manages their diabetic care and patients whose diabetes is managed by a specialist rely upon their PCP for supervising their complete care [7]. Promotion of diabetes management is, in part, dependent upon patient adherence with medical appointments with their PCP. When a patient misses an appointment without canceling a no-show occurs. No-shows to primary care appointments can interrupt continuity of care and effective disease management. Studies report that no-show rates for patients with diabetes range from 4% to 40% [8-13]. In addition, literature indicates that diabetic patients with higher no-show rates have higher glycosylated hemoglobin (A1C) levels and therefore, poorer glycemic control than those patients that attended appointments [8,11,13-16].

Diabetes is one of several chronic conditions that are considered ambulatory care sensitive conditions. Ambulatory care sensitive conditions are those that if treated in a timely and effective manner in the ambulatory care setting should not require acute care services [17]. Use of acute care services for ambulatory care sensitive conditions is considered evidence of a breakdown in outpatient care [18]. A study of hospital claims data from a nationally representative sample of Medicare recipients with diabetes revealed that seven percent of hospitalizations were for diagnoses that have been characterized as ambulatory care sensitive conditions [18]. Similarly, four percent of ED visits are for diagnoses associated with ambulatory care sensitive conditions [19]. To our knowledge, no prior studies have assessed whether no-shows to primary care appointments are associated with increased risk of subsequent ED visits or hospital admissions.

The hypothesis underlying this study is that diabetic patients who no-show to primary care appointments have higher risk for subsequent ED visits and hospital admissions than those who do not miss their scheduled primary care visits. A secondary hypothesis is that compared to patients who attended their last appointment, patients who no-show would be more likely to have admission diagnoses for their subsequent acute care admissions that are sensitive to diabetes-associated ambulatory care.

## Methods

### Study Design

A prospective cohort study was conducted to examine ED visits and hospital admissions six months following the last scheduled primary care appointment. This study was approved by the Institutional Review Boards of Indiana University School of Medicine and Purdue University.

### Patient participants

Patients attending outpatient clinics associated with an academic medical center in Indiana were included in the sample. The sample consisted of 9,411 patients with two or more billing records with International Classification of Diseases (ICD-9) diabetes codes (250.xx, 357.2, 362.0, and 366.41). Only adults, 18 years of age or older, with the above ICD-9 codes were included in the sample (N = 9,393). Diabetic patients with one or more scheduled appointments within the last 2 years (N = 9,387) were included if they had clinical information in the medical records database one year prior to and one year after the last scheduled primary care appointment (N = 8,787).

### Data sources

Patient demographics, diagnoses, appointment history and characteristics, insurance and billing data were retrieved from scheduling and billing data collected during primary care visits occurring between January 2005 and June 2007. Hospital admission dates, emergency department visit dates, and primary diagnoses from January 2005 through to December 2007 were retrieved from the Regenstrief Medical Records System (RMRS) which covers over 1.5 million patients in the greater Indianapolis area. RMRS is utilized by three hospital systems associated with the medical clinics incorporated in this study, including the largest hospital system in Indianapolis.

### Independent variables

In this study no-show status was defined as patients who did not show for their last scheduled primary care appointment. Patients who attended their last primary care appointment comprised the referent group.

### Covariates

Gender was included with female as the referent group. Age was categorized into four ranges: 18–30 years, 31–45 years, 46–70 years, and ≥ 70 years with 18–30 years of age designated as the referent group. Race was classified as black, white, other, and unknown with white as the referent group. Patient insurance coverage was categorized as Medicaid, Medicare, self-pay, county tax-funded program, and commercial insurance with commercial as the referent group. Cardiovascular complications from diabetes included atherosclerosis, angina pectoris, myocardial infarction, heart failure, aortic aneurysm/dissection, other ischemic heart disease (IHD) and other chronic IHD (ICD-9 codes, respectively: 440.xx, 413, 410, 428, 441, 411, and 414) [20]. Nephropathy complications from diabetes included diabetic nephropathy, acute glomerulonephritis, nephritic syndrome, hypertension nephrosis, chronic glomerulonephritis, nephritis/nephropathy, chronic renal failure, renal failure not otherwise specified (NOS), and renal insufficiency (ICD-9 codes, respectively: 250.4, 580, 581, 581.81,582, 583, 585, 586, 593.9) [20]. A Charlson co-morbidity score was computed to describe the number and severity of co-morbidities [21]. To compute this score a weight was applied to each co-morbidity, weights are founded on one-year mortality, and the weighted co-morbidities are totaled for each patient. Charlson co-morbidity scores can range from 0 to 27 with most patients falling below 3. ED visits and hospital admissions in the six months prior to the last scheduled primary care appointment were included, with the referent groups being no ED or hospital admissions in the six months prior, respectively.

### Dependent variables

In one model the dependent variable was time to ED visits within six months (182 days) following the patient’s last scheduled primary care appointment. In the other model the dependent variable was time to hospital admissions within six months after the patient’s last scheduled primary care appointment.

In an analysis addressing the secondary hypothesis, the dependent variables was whether or not the primary diagnosis the first hospital admission was for diabetes diagnoses described in prior publications as potentially preventable. For example, diagnoses included diabetes without mention of complication and diabetes with mention of ketoacidosis, hyperglycemia, hypoglcemia, coma, or an unspecified complication (ICD-9=250.0–250.3, 250.8–250.10, 250.12, 250.13, 250.20, 250.22, 250.23, 250.30, 250.32, 250.33, 250.90, 250.92, or 250.93) [19].

### Statistical analyses

Data analyses were conducted using SAS 9.2. Poisson regression models were computed to determine the bivariate association between each independent variable and each dependent variable. Independent variables with a p-value of 0.20 or less were included in the multivariable models. The Andersen-Gill formulation of the Cox proportional hazard model was used to model the association between no-show at the last scheduled primary care appointment and ED or hospital utilization after adjustment for covariates related to ED or hospital utilization [22]. Unlike the traditional Cox proportional hazard models, the Andersen-Gill model accommodates the dependence between multiple event times that occur when a subject has more than one event (e.g. more than one hospital admission within six months) [22]. There are three different scenarios for calculating time to event (ED visits or hospital admissions). In the first scenario, there is no event and the time interval is defined as six months following the last scheduled primary care appointment. In the second scenario, there is one event within the 182 days following the last scheduled appointment. In this scenario the time interval is defined as days following the last scheduled primary care appointment up to and including the visit/admission date. In the third scenario, there are multiple events and the first time interval is defined as in scenario two above with subsequent time intervals beginning with the previous discharge date and extending to the date of the next visit/admission or the end of the observed interval. Intervals are discontinuous for hospital events because when subjects are hospitalized, they are not at risk of another admission until they have been discharged. A sandwich variance estimator was used to adjust the standard error estimates to account for the dependence among the repeated events within one subject (e.g. multiple ED visits). Each model included the interaction between utilization of the acute care service in the prior six months and no-show status.

In analyses to address whether the acute care admission was for an ambulatory care sensitive condition, a chi-square test was computed to examine the association between no-show status at the last scheduled appointment and whether or not the primary diagnosis was among the set considered to be a potentially preventable diabetes related diagnosis.

## Results

**Figure 1:**
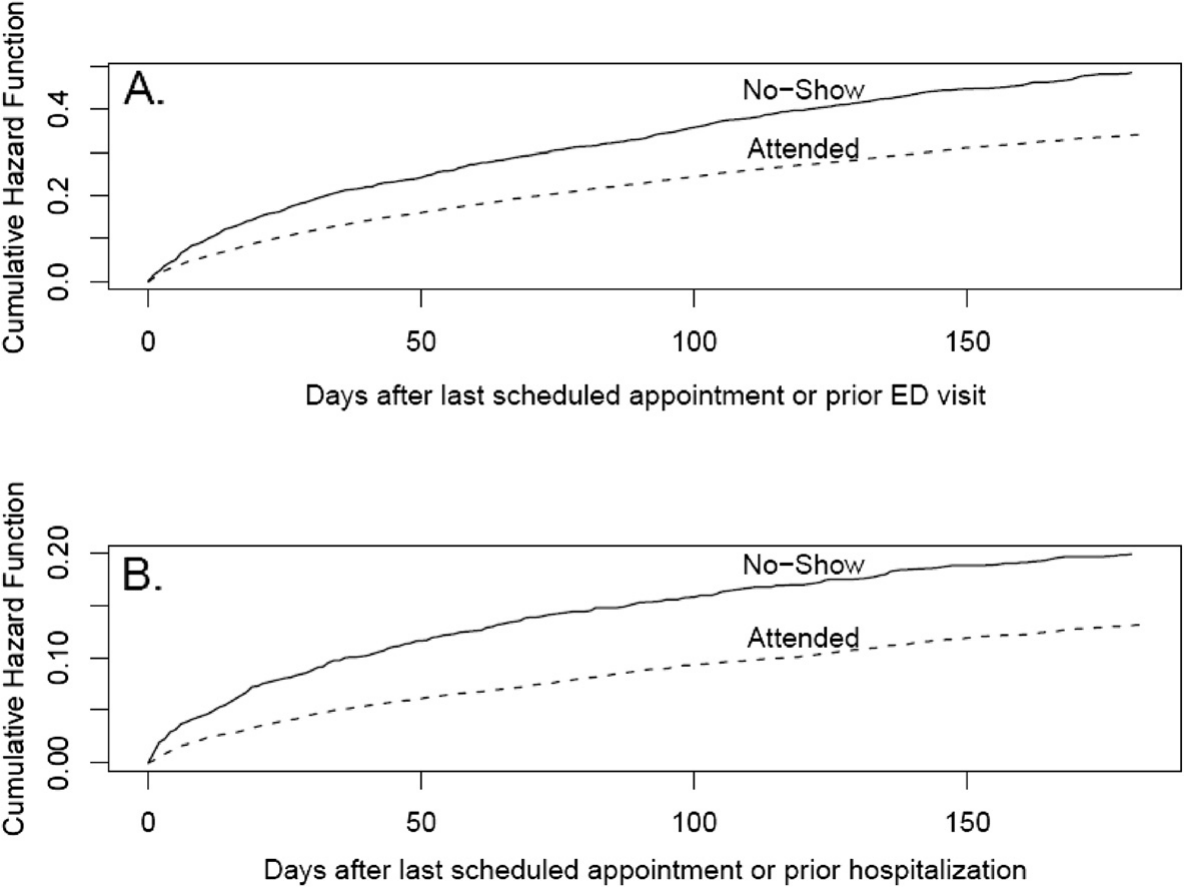
ED visits and Hospital admissions for diabetic patients within 6 months following the last scheduled primary care appointment.

Among the 8,787 patients included in this study, 1421 (16.2%) did not show up to their last scheduled medical appointment. Table 1 reveals that the six-month rate of ED visits following the last scheduled primary care appointment was 0.56 (s.d. = 1.48) for no-shows and 0.38 (s.d. = 1.05) those who attended (*p* < 0.0001). The six-month rate of hospital admissions following the last scheduled primary care appointment was 0.22 (s.d. = 0.83) for no-shows and 0.14 (s.d. = 0.63) for those who attended (*p* < 0.0001). Figure 1 shows that no-show status is associated with time to utilization of acute care services.

**Table 1:** Bivariate analysis on ED visits or hospital admissions within 6 months after last scheduled primary care appointment

**N = 8787**		**N (%)**	**ED visits in**	*P*value*	**Hospital admissions**	*P*value*
			**6 months**		**in 6 months**	
			**mean (SD)**		**mean (SD)**	
**Gender**	**Female**	5268(59.9%)	0.43(1.16)	<0.0001	0.16(0.75)	0.03
	**Male**	3519(40.1%)	0.36(1.09)	-	0.14(0.52)	-
**Age**	**18–30**	314(3.6%)	0.68(1.42)	<0.0001	0.13(0.49)	0.005
	**31–45**	1676(19.1%)	0.56(1.56)	<0.0001	0.12(0.48)	<0.0001
	**46–70**	5695(64.8%)	0.36(1.01)	0.28	0.15(0.73)	<0.0001
	**≥ 70**	1102(12.5%)	0.33(0.80)	-	0.21(0.62)	-
**Race**	**Black**	3655(41.6%)	0.50(1.22)	<0.0001	0.17(0.65)	0.01
	**Other**	782(8.9%)	0.28(0.98)	0.001	0.13(1.21)	0.11
	**Unknown**	383(4.4%)	0.18(0.56)	<0.0001	0.08(0.45)	0.0004
	**White**	3967(45.2%)	0.36(1.11)	-	0.15(0.54)	-
**Insurance**	**Medicaid**	779(8.9%)	0.74(1.88)	<0.0001	0.27(1.00)	<0.0001
	**Medicare**	2746(31.3%)	0.41(1.03)	<0.0001	0.21(0.63)	<0.0001
	**Self-pay**	471(5.4%)	0.47(1.49)	<0.0001	0.11(0.39)	0.04
	**County**	2792(31.8%)	0.46(1.16)	<0.0001	0.13(0.78)	<0.0001
	**Tax-funded**					
	**Program**					
	**Commercial Insurance**	1999(22.8%)	0.17(0.56)	-	0.08(0.35)	-
**Cardiovascular**	**Yes**	1313(14.9%)	0.53(1.30)	<0.0001	0.32(0.74)	<0.0001
	**No**	7474(85.1%)	0.38(1.10)	-	0.13(0.65)	-
**Nephropathy**	**Yes**	526(6.0%)	0.63(1.43)	<0.0001	0.46(1.66)	<0.0001
	**No**	8261(94.0%)	0.39(1.11)	-	0.13(0.54)	-
**Charlson Score**	**1**	6495(73.9%)	0.34(1.00)	<0.0001	0.10(0.50)	<0.0001
	**2**	1497(17.0%)	0.53(1.46)	<0.0001	0.20(0.61)	<0.0001
	**3+**	795(9.1%)	0.68(1.37)	-	0.47(1.44)	-
**ED visits within 6 months prior to last scheduled primary care appointment**	**Yes**	2584(29.4%)	0.86(1.75)	<0.0001	-+	-
	**No**	6203(70.6%)	0.21(0.65)	-	-+	-
**Hospital admissions within 6 months prior to last scheduled primary care appointment**	**Yes**	1081(12.3%)	-+	-	0.58(1.52)	<0.0001
	**No**	7706(87.7%)	-+	-	0.09(0.39)	-
**No-show at last scheduled primary care appointment**	**Yes**	1421(16.2%)	0.56(1.48)	<0.0001	0.22(0.83)	<0.0001
	**No**	7366(83.8%)	0.38(1.05)	-	0.14(0.63)	-

*P values are from Poisson regression. + Bivariate associations for variables that were not to be included in the multivariable analyses were not computed.

Patient characteristics that were significantly associated with the greater risk for subsequent acute care utilization (Table 1) included: female gender; aged 45 or younger; black race; non-commercial insurance; cardiovascular co-morbidity; nephropathy co-morbidity; higher Charlson score; having an ED visit or hospital admission within six months prior to the last scheduled primary care appointment, and no-show at the last scheduled primary care appointment. Table 2 reveals that patient characteristics associated with no-show to the last appointment include younger age, non-white race, Medicaid insurance or self-pay, and acute care utilization in the prior six months.

**Table 2:** Bivariate analysis on no-show status and other covariates

**N = 8787**		**N (%)**	**No-show to last**	*P*value*
			**scheduled appointment**	
			**N (%)**	
**Gender**	**Female**	5268(59.9%)	827(15.7%)	0.14
	**Male**	3519(40.1%)	594(16.9%)	
**Age**	**18–30**	314(3.6%)	87(27.7%)	<0.0001
	**31–45**	1676(19.1%)	359(21.4%)	
	**46–70**	5695(64.8%)	837(14.7%)	
	**≥ 71**	1102(12.5%)	138(12.5%)	
**Race**	**Black**	3655(41.6%)	647(17.7%)	<0.0001
	**Other**	782(8.9%)	172(22.0%)	
	**Unknown**	383(4.4%)	67(17.5%)	
	**White**	3967(45.2%)	535(13.5%)	
**Insurance**	**Medicaid**	779(8.9%)	169(21.7%)	<0.0001
	**Medicare**	2746(31.3%)	365(13.3%)	
	**Self-pay**	471(5.4%)	135(28.7%)	
	**County**	2792(31.8%)	498(17.8%)	
	**Tax-funded**			
	**Program**			
	**Commercial Insurance**	1999(22.8%)	254(12.7%)	
**Cardiovascular**	**Yes**	1313(14.9%)	200(15.2%)	0.32
	**No**	7474(85.1%)	1221(16.3%)	
**Nephropathy**	**Yes**	526(6.0%)	83(15.8%)	0.80
	**No**	8261(94.0%)	1338(16.2%)	
**Charlson Score**	**1**	6495(73.9%)	1064(16.4%)	0.38
	**2**	1497(17.0%)	242(16.2%)	
	**3+**	795(9.1%)	115(14.5%)	
**ED visits within 6 months prior to last scheduled primary care appointment**	**Yes**	2584(29.4%)	553(21.4%)	<0.0001
	**No**	6203(70.6%)	868(14.0%)	
**Hospital admissions within 6 months prior to last scheduled primary care appointment**	**Yes**	1081(12.3%)	288(26.6%)	<0.0001
	**No**	7706(87.7%)	1133(14.7%)	

*P values are from chi-square test.

Results from the Andersen Gill multiplicative hazard model revealed that no-show status was not associated with subsequent ED use after adjustment for covariates related to no-show status and ED utilization. Results from the Andersen Gill model revealed that the interaction between prior hospital admission and no-show on subsequent hospital admissions was significant (*p* = 0.0017). Table 3 shows that compared to patients without a prior hospital admission and attended their appointment, those who had a prior admission and no-showed had the highest risk for subsequent hospitalization (HR = 6.13; CI = 4.60–8.18), followed by those with a prior hospitalization, but attended their last appointment (HR = 3.84, CI = 3.01–4.90).

Among the subgroup of diabetic patients who had a prior hospital admission, those who no-showed were at 60% greater risk for subsequent hospital admission (HR = 1.60, CI = 1.17-2.18) than those who attended their appointment. No-shows who did not have a prior admission were not at greater risk than patients without a prior hospital admission and attended their appointment (HR = 0.83, CI = 0.63–1.09). No-shows were significantly more likely to have hospital admission for diabetes diagnoses described in prior publications as potentially preventable compared to those that attended their appointment (Table 4: 13.64% versus 4.37% respectively; Chi-square = 20.47; dF = 1; *p* < = 0.001).

**Table 3:** Hospital admissions within 6 months after last scheduled primary care appointment

**Parameter**		**Chi-**	**Pr >**	**Hazard**	**95% Hazard**
		**Square**	**ChiSq**	**Ratio**	**Ratio**
					**Confidence**
					**Limits**
**Gender**	**Male vs. Female**	0.12	0.72	0.97	0.82–1.15
**Age**	**31–45 vs. 18–30**	0.29	0.59	0.89	0.59–1.35
	**46–70 vs. 18–30**	0.02	0.88	0.97	0.64–1.48
	**≥ 71 vs. 18–30**	0.007	0.93	0.98	0.62–1.55
**Race**	**Black vs. White**	0.39	0.53	1.05	0.90–1.24
	**Other vs. White**	0.27	0.61	1.18	0.63–2.23
	**Unknown vs. White**	3.85	0.05	0.57	0.33–1.00
**Insurance**	**Medicaid vs. Commercial**	21.70	<0.0001	2.21	1.58–3.09
	**Medicare vs. Commercial**	15.19	<0.0001	1.67	1.29–2.16
	**Self-Pay vs. Commercial**	1.85	0.17	1.31	0.89–1.91
	**County Tax-funded Program vs. Commercial**	11.37	0.0007	1.55	1.20–2.00
**Cardiovascular**	**Yes vs. No**	6.15	0.01	1.32	1.06–1.65
**Nephropathy**	**Yes vs. No**	5.29	0.02	1.40	1.05–1.86
**Charlson Score**	**2 vs. 1**	3.21	0.07	1.22	0.98–1.52
	**≥ 3 vs. 1**	29.22	<0.0001	2.05	1.58–2.66
**Status at last scheduled primary care appointment and prior hospital admissions within 6 months**	**Arrived and no prior**	-	-	-	-
	**hospital admission**				
	**Arrived and prior**	117.01	<0.0001	3.84	3.01–4.90
	**hospital admission**				
	**No-showed and no prior hospital admission**	1.84	0.17	0.83	0.63–1.09
	**No-showed and prior hospital admission**	152.09	<0.0001	6.13	4.60–8.18

**Table 4:** Hospital admissions within 6 months following the last scheduled primary care appointment and whether the primary diagnosis was for diabetes diagnoses described in prior publications as potentially preventable

	**Diabetes diagnosis (No)**	**Diabetes diagnosis (Yes)**	**Total**
**Attended**	656(95.6%)	30(4.4%)	686(79.6%)
**No-show**	152(86.4%)	24(13.6%)	176(20.4%)
**Total**	808(93.8%)	54(6.3%)	862(100%)

* *p* < 0.0001 from chi-square test.

## Discussion

This study provides evidence that among diabetic patients with a recent history of a hospital admission, those that no-show to their PCP appointment are at significantly greater risk for subsequent ED visits and hospital admissions than those that attended their PCP appointment. This finding confirms and extends prior research that describes the potential health consequences of patients not showing to their primary care appointment. Prior studies reported that those patients that missed their primary care appointments had poorer glycemic control [8,11,13-16,23]. However, prior studies have not reported that patients with diabetes who miss their medical appointments are at increased risk for subsequent hospital admissions. No-shows can disrupt continuity of care which is vital for maintenance and improvement of diabetics’ health status >[24]. Results from this study provide evidence of the need for proactive interventions that significantly reduce no-show rates.

Acute care utilization may reflect a substitution of acute care services for primary care services for some patients [25]. A recent study revealed patients that no-showed to their primary care appointments were significantly more likely to receive their diabetes care using same-day appointments [23]. This suggests that patients that no-show may be more reactive than proactive in their approach to managing their diabetes [23]. A reactive approach is particularly problematic for patients with a recent history of hospitalization as it does not allow the opportunity for continuous treatment and monitoring of the condition that precipitated the hospital admission. This study provides indirect evidence that interruptions in primary care can contribute to poor management of diabetes because, compared to patients who attended their appointment, patients that no-showed were more likely to be admitted for diabetes diagnoses described in prior publications as potentially preventable.

Clinic-based interventions to reduce no-shows include telephone reminders which only modestly decrease no-show rate [26-28] and open access scheduling [29]. Although open access scheduling significantly reduces no-show rates, it does not adequately support chronic care because the patient has to initiate the next appointment; that is, the patient has to remember to call for an appointment at the appropriate time as specified in clinical practice guidelines. Future clinic-based interventions to reduce no-show rates should consider additional modifiable clinic procedures that influence no-show rate. For example, our prior work demonstrated that patients who schedule their visit more than two weeks in advance are twice as likely to no-show to their next scheduled appointment [30]. An automated system that reminds patients to make appointments within weeks of their next needed appointment may be effective in reducing no-show rate.

Future development of clinic interventions to reduce no-show rate should consider costs of no-shows compared to costs associated with developing a multi-factorial approach that includes proactive planning, scheduling, reminding and rescheduling when patients miss an appointment. Costs of no-show extend beyond lost revenue for the missed appointments [30]. This study provides evidence that costs of no-show should also include downstream costs associated with the cost of acute care that occurs after no-shows. An average charge for a hospital stay for a diabetic is nearly $11,000 [3]. The 1421 no-shows in this study experienced an excess of 95 hospital admissions compared to subjects that attended their last medical appointment. This estimate was calculated by multiplying the difference in admission rates between the attended/no-show groups to the total number of no-shows. Other potential costly consequences include potential duplication of services such as laboratory and radiology tests. Clearly, these costs provide impetus for the development of a multi-component intervention that can be implemented in the clinic setting.

The results of the study must be considered in the context of its limitations. The subjects from this study were patients enrolled in a Midwestern, urban, university-associated primary care medical system. The demographic characteristics of this sample are similar to those of a national sample of patients with diabetes who attended academic medical centers and thus, generalizability of results may be limited to similar primary care settings [31]. The unavailability of data about the purpose of the visit appears to be a universal problem across no-show studies. No-showing to one visit is not an indicator of long-term appointment keeping behavior; however, it does provide evidence that a snapshot of no-show behavior is predictive of future acute care utilization. Furthermore, it is not possible to describe the association between no-show and medication compliance or glycemic control because not all patients’ pharmacy and lab information were integrated into the medical records database. Thus, we cannot assess whether the association between no-show behavior and subsequent acute care utilization could be explained by poor glycemic control.

This study does not capture ED visits and hospital admissions that did not occur at one of the hospitals or emergency departments associated with the three hospital systems included in the RMRS. Thus, out-of-town admissions were not included and this may have resulted in underestimation of hospital and emergency department utilization rates. This descriptive study from a clinical setting is not designed to make statements about causation. Specifically, we cannot address whether low health literacy could have explained the association between no-show status and subsequent acute care utilization. Furthermore, we were not able to describe whether there was a recursive association between no-show status and hospital admissions. By statistically adjusting for hospital admissions in the prior six months, we may have underestimated the association between no-show behavior and subsequent hospital admissions. Nonetheless, the findings provide strong evidence that among diabetics with a recent hospital admission, those who no-show are at increased risk for a future hospital admission.

## Conclusions

The problem of no-shows has long been considered an outpatient clinic operations problem that disrupts providers’ care plans for their chronic care patients. This study reveals that outcomes of patients who no-show extend beyond the outpatient clinic setting because patients that no-show have significantly higher rates of subsequent acute care utilization. The results provide evidence of the importance of proactive re-scheduling of patients who no-show because of their vulnerability for future hospital admissions.

## Competing interests

The authors declare that they have no competing interests.

## Authors’ contributions

LN, LS, ML, AT and ZT carried out the study, participated in data analysis and interpretation, drafted and revised the manuscript. ZT and LZ were responsible for the statistical analyses. LZ, KC and DW were involved with drafting and revising the manuscript. All authors read and approved the final manuscript.

## Pre-publication history

The pre-publication history for this paper can be accessed here:

http://www.biomedcentral.com/1472-6963/12/304/prepub
